# Experiences of a Mindfulness-Based Telehealth Program Modified for Adults with Cerebral Palsy—A Qualitative Study

**DOI:** 10.3390/healthcare14020197

**Published:** 2026-01-13

**Authors:** Georgina Henry, Ingrid Honan, Emma Waight, Katherine Swinburn, Fiona Given, Sarah McIntyre, Hayley Smithers-Sheedy

**Affiliations:** 1 Cerebral Palsy Alliance Research Institute, Specialty of Child and Adolescent Health, Sydney Medical School, The University of Sydney, Sydney, NSW 2050, Australia; ingrid.honan@cerebralpalsy.org.au (I.H.); emma.waight@cerebralpalsy.org.au (E.W.); kath.swinburn@cerebralpalsy.org.au (K.S.); smcintyre@cerebralpalsy.org.au (S.M.); hsmitherssheedy@cerebralpalsy.org.au (H.S.-S.); 2 UTS Disability Research Network, University of Technology Sydney, Sydney, NSW 2007, Australia; fiona.given@uts.edu.au

**Keywords:** cerebral palsy, mindfulness-based stress reduction, mindfulness, anxiety, emotion regulation, telehealth, group intervention

## Abstract

**Highlights:**

**What are the main findings?**

- Participants valued learning different mindfulness techniques together, guided by an expert facilitator, and practicing them in daily life.
- Participants recommended incorporating an orientation and follow-up sessions before and after the telehealth program and reducing the duration of sessions to minimize the impact of fatigue.

**What are the implications of the main findings?**

- Group telehealth programs for adults with cerebral palsy should be hosted by a skilled group facilitator who has expertise in cerebral palsy.
- Participant recommendations should inform future implementation of group mindfulness telehealth programs for adults with cerebral palsy.

**Abstract:**

**Backgrounds/Objectives:** Mindfulness-based stress reduction (MBSR) programs may have applications for adults with cerebral palsy (CP), particularly as this population is at increased risk of mental health challenges relative to the general population. However, little is known about the experiences of adults with CP participating in these programs. The aim of this study was to explore the experiences of adults with CP, and a facilitator, who participated in a 9-week MBSR telehealth program. **Methods:** Adults who attended an MBSR telehealth program were invited to participate in focus groups. If a participant was unable to attend a focus group, they were offered a semi-structured interview. The facilitator participated in a semi-structured interview. Focus groups and interviews were recorded, transcribed verbatim, and inductively thematically analyzed using Framework Analysis. **Results:** Ten adults with CP and one facilitator participated. Feedback on the program spanned across three themes: (i) learning and creating my mindfulness toolbox; (ii) applying mindfulness to everyday life; and (iii) online together with expert facilitation. Participants appreciated having access to a variety of mindfulness techniques to accommodate individual preferences. Peer-learning in a facilitated, online group context was also valued. Participants recalled implementing mindfulness strategies in everyday life and provided recommendations of how to improve the program. These included incorporating a group orientation, shortening group sessions to reduce fatigue, and follow-up sessions to maintain mindfulness skills after program completion. **Conclusions:** This study provides new knowledge about the perspectives of adults with CP regarding MBSR delivered via telehealth. Participant recommendations should inform future implementation of group mindfulness telehealth programs for adults with CP.

## 1. Introduction

Cerebral palsy (CP) is a lifelong, non-progressive physical disability which affects movement and posture, caused by injury to, or maldevelopment of, the developing brain [1]. People with CP may also experience co-morbidities including epilepsy, intellectual disability, hearing, vision, speech, and feeding impairments [1]. Physical health and rehabilitation have long been the primary focus of CP research and clinical care. However, a growing body of evidence indicates that people with CP are also more likely to experience mental health disorders than the general population [2,3,4]. Relative to population norms, adults with CP have reported elevated symptoms of anxiety and depression [5]. This is likely due to a range of mental health risk factors which are commonly experienced by people with CP across their lifespan, including chronic pain, fatigue, emotion regulation difficulties, discrimination, social exclusion, and disability-related stigma [5,6,7].

Mindfulness-based stress reduction (MBSR) is an intervention which was originally developed for stress and pain management [8]. It has now also been used to manage a variety of psychiatric, psychosomatic, and physical conditions [9,10]. The practice of mindfulness involves cultivating non-judgmental awareness, and acceptance, of present-moment experiences [11,12]. Through continual monitoring of current experiences with openness and curiosity, maladaptive thoughts related to the past and future can become deflated [12]. By improving emotional regulation skills, evidence suggests that MBSR may have wide applications for coping with adversity in a range of different clinical populations [9,10].

As a relatively new area of CP research, only a few studies have explored the potential benefits of mindfulness for people with CP [13]. One randomized control trial (RCT) of a mindfulness-based movement program for children with CP identified short-term improvements in attention levels [14,15]. A pilot study of adults with CP found that an MBSR telehealth program significantly improved pain catastrophizing 4 months post intervention [16]. In a recent RCT examining an MBSR telehealth program for adults with CP, our team identified no significant difference in mindfulness knowledge between groups [17]. However, compared to baseline, the intervention group’s mindfulness and emotion regulation skills significantly improved immediately post intervention and at an 8-week follow-up. Stress and depression symptoms in the intervention group also decreased significantly after the program but were not sustained at follow-up.

In addition to quantitatively evaluating the impact of the MBSR telehealth intervention, understanding participants’ experiences and their recommendations regarding future implementation remains important. The recent literature recommends the use of mixed methods to evaluate online mindfulness interventions for people with physical conditions [18]. Qualitative enquiry may provide insight into how such interventions can be appropriately tailored and implemented to suit the needs and preferences of participants with different abilities and accessibility requirements [18]. To date, with the exception of a small, brief exploration conducted in 2020 [16], there have been no other studies which have investigated the experiences of adults with CP participating in MBSR programs. This is a missed opportunity to gain valuable insights which may assist in improving the quality and accessibility of MBSR telehealth programs for adults with CP. This study aimed to qualitatively explore participants’ and the facilitator’s experiences of an MBSR telehealth program.

## 2. Materials and Methods

### 2.1. Study Design and Participants

This qualitative study was conducted as a component of a two-part sequential evaluation of an MBSR telehealth program for adults with CP.

Part one involved a quantitative evaluation which is published elsewhere (see reference [17] for full details). Inclusion criteria for enrolment into the MBSR program were: adults with CP aged 20–40 years, functional hearing and vision, no or mild intellectual disability (ID) and ability to communicate verbally or used augmentative and alternative communication (AAC). To be eligible to participate, participants also self-identified as having elevated anxiety or emotion regulation difficulties and elevated scores on either the Depression and Anxiety Stress Scales (DASS) or Difficulties in Emotion Regulation Scale (DERS) at baseline assessment [17].

Upon completion of the intervention, MBSR participants were invited to participate in part two—the qualitative evaluation. This paper describes the methods and results of this qualitative evaluation.

### 2.2. The MBSR Program

The 9-week program involved weekly 90 min group telehealth sessions, and self-guided practice activities undertaken between sessions. Each session explored a different topic and included a range of mindfulness techniques (see Table 1). Formal mindfulness exercises involved a structured practice (e.g., sitting meditation), whereas others were informal and could be integrated into everyday activities (e.g., STOP: stop, take a breath, observe, proceed). MBSR program participants were provided with a program manual, home practice activities, exercise sheets, and audio file links to mindfulness meditation resources. Before the program, the facilitator contacted each MBSR program participant individually and practiced accessing Microsoft Teams (version 1.6.00.34637). Between group sessions, the facilitator followed up with MBSR program participants individually if they required further support. If an MBSR program participant missed a group session, they were able to access a recording during the following week.

### 2.3. Participant Recruitment for Qualitative Evaluation

Criterion sampling was used, whereby all participants who had shared experience of taking part in the MBSR telehealth program were invited to participate in the qualitative evaluation. All adults with CP who met the inclusion criteria for the RCT and had completed (n = 30) the MBSR telehealth program were eligible to participate. MBSR participants were invited via email to participate in an online focus group [17]. Email invitations included a participant information statement and a link to an electronic REDCap (Research Electronic Data Capture version 14.1.4) consent form [22,23]. Potential study participants were informed that they would be asked about their experience of the MBSR program and how to improve it for future participants/facilitators.

The MBSR facilitator (n = 1) was invited separately by email to participate in a one-on-one online interview. The facilitator was also provided with a participant information statement and electronic consent form. The facilitator was a social worker who had extensive prior experience working with people with CP, facilitating groups, and had completed MBSR training.

### 2.4. Data Collection: Focus Groups and Semi-Structured Interviews

Focus groups and semi-structured interviews were conducted on Microsoft Teams (version 1.6.00.34637) [17] between July 2022 and December 2022. A deductive approach was adopted for data collection—a topic guide and interview guide were used to explore participants’ experiences of and recommendations for the MBSR telehealth program (Supplementary File S1) [24]. The guides comprised three topic areas: (i) overall experiences of the MBSR program; (ii) feedback on the program content and accessibility; and (iii) impact of the program on MBSR program participants’ everyday life. Focus groups and interviews were video recorded and transcribed verbatim.

If only one MBSR program participant was available for a scheduled focus group time, they were invited to participate in a one-on-one semi-structured interview format, to maximize inclusion [25,26]. The facilitator was interviewed individually and was not present at any of the focus groups or interviews with participants who had completed the MBSR program.

During focus group discussions, efforts were made to enable the inclusion of participants who used AAC (n = 3). For example, participants who used AAC were given the topic schedule questions before the focus groups—to allow time to prepare their answers in their communication systems. During group discussions, participants who used AAC were encouraged to take the time they needed to answer questions. They were also given the opportunity to contribute to group discussion via real-time electronic chat functions (via typing rather than using their AAC voice output system).

### 2.5. Data Analysis

MBSR program participants’ clinical and demographic characteristics were analyzed descriptively. NVivo 13 was used to inductively thematically analyze qualitative data using the framework method as described in Table 2 [24,27,28]. Data were analyzed from a contextualist perspective, whereby knowledge was accessed through participants’ reflections and lived experiences [29]. Data source triangulation was used to compare responses by each MBSR program participant (P) or the facilitator (F) [25]. MBSR program participant and facilitator responses were cross-referenced and similarities and differences between, and within, themes were tracked whilst charting the data (see Table 2). Findings which were specific to a collection method (i.e., focus group vs. interview) were also distinguished to increase the validity of analysis [24]. Researcher triangulation was adopted to increase the trustworthiness of the analysis, whereby all findings were reviewed by a multi-disciplinary team, including a research partner with lived experience of CP (FG) [25]. Reporting was guided by the COREQ-32 checklist [30] and Standards for Reporting Qualitative Research [31].

### 2.6. Transparency and Openness

During data collection and analysis, the authors engaged in team reflexivity. Adopting a postpositivist approach, team reflexivity involved attempts to uncover researcher bias to reflect participants’ true perspectives as closely as possible [32,33]. The team met frequently to discuss and uncover any potential biases we brought as disability academics who quantitatively evaluated the MBSR program [34]. Specifically, coding inconsistencies were discussed by a minimum of 3 researchers until consensus was reached. Similarly, decisions around which data to include and how to include them in reporting were discussed with all members of the authorship team to ensure reporting most accurately reflected participants’ perspectives. All interviews and focus groups were conducted by a trained female qualitative researcher (G.H.) who was not involved in provision of the MBSR program. Member checking was not able to be conducted due to the potential for conflicting interpretations of collective focus group transcripts, and logistical difficulties of ensuring the involvement of all participants [35]. All eligible participants who consented to participate were included in the sample to maximize the chances of achieving data saturation [36]. No authors had any clinical involvement with participants. This study was approved by The University of Sydney Human Research Ethics Committee [Project number: 2021/239].

## 3. Results

Eleven participants took part in this study (10/30 MBSR program participants and one facilitator). Eight MBSR program participants took part in one of two focus groups; two participants were interviewed as they were unable to join the focus groups. The combined mean focus group and interview duration was 69 min (range 44 min to 91 min). MBSR program participants had a range of demographic and clinical characteristics and resided across three different states of Australia (Table 3). The majority (n = 7) were female and the mean age was 26 years (SD = 3.83; range 21–31 years). Most (n = 8) had a predominant motor type of spastic CP and three used AAC to communicate. Most (n = 9) lived in major cities. In addition to CP, half of the MBSR program participants reported a psychological and/or other neurodevelopmental diagnosis.

### 3.1. Themes

Three themes emerged from the thematic analysis: (1) learning and creating my mindfulness toolbox; (2) applying mindfulness to everyday life; (3) online learning together with expert facilitation. These themes are presented below with illustrative quotes identified by source [MBSR program participant (P) or facilitator (F)] and participant ID. All participants contributed data to each of the three themes, indicating inductive thematic saturation [36]. Supplemental quotes from focus groups and semi-structured interviews are included in Table S1 (see Supplementary File S2).

#### 3.1.1. Learning and Creating My Mindfulness Toolbox

Both the MBSR program participants and the facilitator thoroughly enjoyed the mindfulness program, describing it as “valuable” (P1), “fantastic” (P8), “good” (P2), and “fabulous” (F1). MBSR program participants indicated that they would recommend the program to others, highlighting how much they learned.

“I did enjoy the process of learning how to be more mindful”

(P5)

“To be able to understand why, and what your body is doing [*when you feel anxious*]… and have an education behind [*it*], I think it makes it a lot easier…to then work forward”

(P10)

MBSR program participants appreciated having access to a variety of mindfulness techniques to accommodate individual preferences. By trialing different exercises, each MBSR program participant had the opportunity to create their own mindfulness “toolbox” (P9) of strategies. Some MBSR program participants preferred formal mindfulness practices, while others preferred informal practices. Both the MBSR program participants and the facilitator noted that having a selection of exercises with different durations enabled MBSR program participants to choose mindfulness strategies to suit their needs at any given time.

“We got a wide range of audios… if one technique didn’t work for you, then you can go back to one that did”

(P7)

“The best thing about the program was having access to a range of mindfulness exercises, tools that we can use in different situations”

(P9)

The facilitator observed a general preference for “shorter” (F1) exercises, with an interviewee explaining these suited their attention span. These techniques were “quick and concise…able to do anywhere” (P10).

MBSR program participants discussed some of the challenges experienced during the program and reported difficulties either remembering or overthinking when to engage in the home practice (P3, P8). Some did not like the audio features used in the guided mindfulness exercises. For example, one MBSR program participant and the facilitator found it off-putting that some recordings ended with a “really loud” (P5, F1) bell. Others did not find the recorded voices relaxing to listen to (P5, P7). Listening to long pauses during the meditation recordings also reportedly made some feel uncomfortable (P4, P6, P7).

“[*I*] kept forgetting to do the daily activities and I found overthinking when…to do it [*practice*]”

(P3)

“They’d be pauses [*during the meditation recording*], and it was weird for me to sit, and I’d be like, ‘when is he going to say something next?’”

(P4)

#### 3.1.2. Applying Mindfulness in Daily Life

Overall, MBSR program participants reported that the mindfulness program had a positive effect on their psychological wellbeing. MBSR program participants indicated the program was helpful for managing anxiety, with two interviewees (P7, P10) attributing this to an enhanced ability to identify their feelings and implement management strategies. The facilitator noted that throughout the program, MBSR program participants frequently spoke of “feeling less anxious” and would notice things “in their lives that were positives” (F1). MBSR program participants also described having gained an increased sense of self-awareness (P5, P7).

“I’ve personally benefited a lot from doing it [*the mindfulness program*]”

(P9)

“The whole program helped me manage my anxiety better”

(P2)

“It’s [*the mindfulness program*] made a major impact in knowing myself a bit better… noticing those changes in my body that indicate that I’m stressed…I never would have picked up on before”

(P7)

MBSR program participants recalled using techniques taught in the mindfulness program to help manage their emotions. A few mentioned using short techniques either during anxiety-inducing situations (e.g., in a doctor’s waiting room) or when needing to focus (P1, P6, P10). Another spoke of using guided meditations to help “wind down” (P10) before bed.

The facilitator also disclosed anecdotes that MBSR program participants had shared during the MBSR program. For example, some found one activity “really helpful” (F1) for managing pain. Others shared used mindfulness strategies when sick in bed to mitigate feeling “really down” (F1). The facilitator also recalled that one MBSR program participant, during a group session, had described practicing mindful eating and how it helped with their ability to manage their meal safely.

MBSR program participants also discussed when practicing mindfulness was most impactful. Three reported that using mindfulness strategies helped to prevent anxiety (P2, P5, P7).

“I find it easier to concentrate on mindfulness if I’m not in the middle of anxiety…. before I get to the point of an anxiety attack”

(P2)

Others found it helpful to practice mindfulness when they were already feeling anxious (P4).

Both the facilitator and MBSR program participants reflected on other perceived benefits which they linked to the mindfulness program. The facilitator recalled one MBSR program participant (who used a wheelchair) was “able to manage their anxiety to the extent that they were able to catch a lift [*elevator*]” (F1) independently. Other MBSR program participants mentioned having an increased ability to go outside, spend more time with family and friends, and improved sleep quality (P6, P8).

#### 3.1.3. Online Together with Expert Facilitation

Overall, MBSR program participants enjoyed the program structure and commented that nine weeks was an appropriate length. MBSR program participants suggested follow-up sessions could be offered post completion to assist with maintaining mindfulness skills. MBSR program participants liked the “ease of access” (P10) of the online format. One who used a wheelchair for mobility described the convenience of not having to organize transport to attend a face-to-face meeting (P9). Another who was interviewed noted that the online format allowed flexibility when juggling work commitments (P7). Two MBSR program participants also found the catch-up session recordings very useful if they missed a session (P7, P9); others forgot they were able to access these as they were not reminded.

“It [*the online format*] was good for me because I wouldn’t have been able to get somewhere every week”

(P9)

Both the MBSR program participants and the facilitator highlighted that group learning was “really valuable” (P9). The ability to share learnings “really validated” (P7) MBSR program participants’ experiences. Both the MBSR program participants and the facilitator indicated that six to eight group members would be an ideal group size, allowing sufficient opportunity and comfort to share.

MBSR program participants said that they would have benefited from an initial meet-and-greet group orientation to dispel nerves about attending the group, and to discuss how best to include/support group members who used AAC. Nonetheless, one MBSR program participant who used AAC felt the group “did a good job accommodating” (P3) them. The facilitator also observed that many MBSR program participants were “really generous to one another support-wise” and “enjoyed hanging out with other people with CP” (F1). One MBSR program participant who was interviewed would have liked the “opportunity to…make friends” and connect with other group members outside of the group sessions (P7).

Overall, most MBSR program participants recommended the session duration be shortened from 90 min to 1 h to help prevent fatigue. Both the MBSR program participants and the facilitator enjoyed commencing the weekly sessions by reviewing participants’ progress with the home practice exercises. However, two MBSR program participants explained that this meant other content was sometimes rushed (P1, P5).

MBSR program participants highlighted the benefits of being able to check in with an experienced facilitator during weekly sessions—to ask questions and troubleshoot any challenges. MBSR program participants valued the empathetic style of the highly skilled facilitator, describing her as “calm” (P5) and “really friendly and inclusive” (P9).

“I think [*the best thing about the mindfulness program was*] the opportunity to…have someone like [*the facilitator*], to be able to talk to and ask questions”

(P10)

The facilitator shared insights about program delivery. The facilitator felt it was important to have knowledge about CP, as participants would frequently “talk about CP-related challenges” (F1). Additionally, expertise in running groups, and an understanding of how anxiety and depression can impact individuals were necessary components of facilitation. The facilitator would recommend the program to future facilitators.

## 4. Discussion

This qualitative study explored the experience of individuals who participated in, or facilitated, an MBSR telehealth program modified for adults with CP. Overall, MBSR program participants perceived the program to be informative, accessible, and enjoyable. MBSR program participants reported the program positively affected their psychological wellbeing. MBSR program participants valued the accessible online group format and highlighted the benefits of learning from a highly skilled facilitator. Recommendations about how to improve the future implementation of the program were also discussed.

MBSR program participants indicated the telehealth MBSR program provided them with helpful tools and strategies for managing emotions in various situations. This is consistent with the quantitative evaluation which found that compared to baseline, the intervention group had improved mindfulness, emotion regulation skills, and reduced stress and depression immediately post intervention [17]. In this qualitative study, some MBSR program participants identified that mindfulness techniques were helpful for managing their anxiety. While this was not reflected in the quantitative evaluation [17], this does align more consistently with a wider body of the mindfulness intervention literature. Extensive research demonstrates that mindfulness interventions can have small to large effects on reducing elevated symptoms of anxiety and depression across a wide range of clinical populations [9,40,41,42].

Some MBSR program participants attributed a positive effect on wellbeing to an increased sense of self-awareness and an improved ability to identify their feelings. This is consistent with existing qualitative research in which some adults with CP or other chronic health conditions have explained mindfulness interventions have helped them to stay calmer in stressful situations, increase acceptance, improve self-awareness, and cultivate positive feelings [16,43]. These sentiments have been reported in survey data which indicated increased mindfulness skills may be protective against anxiety and depression in people who experience chronic pain [44,45]. Mediation analyses have also identified that improved emotion regulation skills, such as increased levels of self-compassion, and awareness of internal and external experiences, may explain why mindfulness interventions can reduce symptoms of anxiety in people living with physical health conditions [46,47,48].

To optimize the impact on participants’ wellbeing, MBSR telehealth programs must be accessible and acceptable to adults living with CP. The MBSR program participants in our study resided across three different Australian states (as far as 1800 km apart), with the majority attending from major cities. Many valued the accessible online format, with some commenting this made it possible for them to participate. The practical convenience of telehealth minimizes geographical and transport barriers, reduces energy expenditure, and makes attendance easier [16,49]. Telehealth interventions may also be cost-effective, especially for people with substantial disability-related out-of-pocket costs [50,51].

Group learning was generally perceived as validating and enabled MBSR program participants to connect with others who had similar experiences. Group learning has been shown to strengthen social connectedness and heighten self-compassion in a wide range of clinical populations [13,16,43,52]. The desire for social connection, aligns with research revealing that adults with CP may encounter obstacles forming relationships due to disability-related stigma [7]. This indicates that group interventions may have a role to play in combating social isolation.

MBSR program participants also discussed how helpful it was to follow up weekly with a highly skilled facilitator. Facilitators can provide invaluable support to help manage group dynamics whilst also allowing individuals to access appropriate adjustments and follow-up as needed [17,43]. These are worthwhile considerations—as mindfulness is a relatively new area of CP research, there may be an increased need for professionals with appropriate expertise in both CP and MBSR [13]. Overall, MBSR program participants recommended the session duration be reduced to one hour. This finding is consistent with research indicating that the standard duration and frequency of online MBSR programs may need to be modified for people with reduced physical function (e.g. multiple sclerosis) [18]. MBSR program participants also indicated an initial orientation to the group would have helped to dispel nerves. Future programs could also consider adding follow-up sessions post completion to support maintenance of skills [13,43].

This study has several strengths and some limitations. As MBSR program participants self-selected to participate in both the MBSR program and the qualitative interviews, selection biases may have been present. While the experiences of MBSR program participants who chose not to take part in the qualitative study remain unknown, those who did participate had a range of gross motor and communication abilities. Future qualitative studies involving participants who use AAC should consider embedding additional alternative adaptations to further improve participation, for example, offering multiple interviews or focus groups, or modifying response options [53]. Future research should also investigate the MBSR telehealth program experiences of adults with CP who were not eligible to participate in this program, i.e. individuals with intellectual impairment, sensory impairments, and those who do not identify as having existing emotion regulation difficulties.

## 5. Conclusions

This qualitative study highlights that participants enjoyed the modified mindfulness program for adults with CP. MBSR program participants valued learning different mindfulness techniques together, guided by an expert facilitator, and practicing them in daily life. Participant recommendations provided in this study should inform future implementation of mindfulness group telehealth programs for adults with CP. These include the following: (1) incorporating an orientation before programs; (2) ensuring program materials are accessible and offer diverse content to accommodate individual preferences; (3) ensuring the length of telehealth sessions minimizes the impact of fatigue on participants’ learning; (4) including follow-up sessions to help maintain skills after program completion.

## Figures and Tables

**Table 1:** Overview of 9-week mindfulness-based stress reduction program.

Week	Mindfulness Session	Mindfulness Technique
1	Course overview and what is mindfulness?	Mindful check-in [19]
2	The mind/body connection: how our body responds physiologically to anxiety and stress	Grounding exercise [20]
3	How to practice mindfulness meditation	8 attitudes for mindfulness [19]
		Mindful breathing [19]
		Mindful Word for dealing with “wandering mind” [19]
4	How mindfulness works with anxiety and stress	ACT: accept, choose, take action [21]
		Mind Traps [21]
		Riding the storm out [21]
		STOP: stop, take a breath, observe, proceed [19]
5	Mindfulness for the body	Minding your pain [19]
		The Body Scan [19]
6	Deepening your practice—sitting meditation	Sitting meditation [19]
7	Meditation for anxiety and stress	Mindful self-inquiry [19]
		Turning into emotions [19]
		RAIN: recognize, allow/accept, investigate, non-identify [19]
8	Loving kindness meditation	Loving kindness meditation [21]
9	Interpersonal mindfulness and how to make mindfulness part of your everyday life	Interpersonal mindfulness qualities [19]
		Creating Connection [21]

**Table 2:** Stages of framework analysis.

Framework Analysis Stage	Description
Familiarization with the data	Five researchers (G.H., I.H., E.W., H.S.S., and K.S.) independently read the transcripts to become familiar with the data.
Developing a coding framework	Key concepts were used to develop an initial coding framework.
Indexing	The coding framework was systematically applied to all transcripts.
Charting	All coding inconsistencies were discussed, and the coding framework was iteratively amended.
Mapping and interpretation	Overarching relationships and patterns within the data were identified.

**Table 3:** Demographic and clinical characteristics of MBSR program participants.

Demographic/Clinical Characteristics	MBSR Program Participants, n (%)
Age (years)	
21–25	5 (50)
26–31	5 (50)
Sex	
Male	3 (30)
Female	7 (70)
Employment	
Employed/Student	5 (50)
Not employed	5 (50)
Cerebral Palsy Predominant Motor Type and Topography	
Unilateral spastic cerebral palsy	1 (10)
Bilateral spastic cerebral palsy	7 (70)
Other *	2 (20)
Gross Motor Function Classification System (GMFCS) ^1^ [37]	
Level I–III	5 (50)
Level IV-V	5 (50)
Communication Function Classification System (CFCS) ^2^ [38]	
Level I	6 (60)
Level II–V	4 (40)
Augmentative/Alternative Communication System Used	
Yes	3 (30)
No	7 (70)
Viking Speech Scale (VSS) ^3^ [39]	
Level I	5 (50)
Level II–IV	5 (50)
Additional Neurodevelopmental/Psychological Disorder **	
Yes	5 (50)
No	5 (50)

^1^ The GMFCS describes five levels of gross motor abilities for people with CP. Levels I–III describe people who may have balance and coordination challenges, to those who require the use of hand-held mobility devices. Levels IV–V describe people who require physical assistance or powered mobility devices in most settings, to those who have difficulty maintaining antigravity head and trunk postures. ^2^ The CFCS describes five levels of communication abilities for people with CP. Level I describes people who can effectively send and receive information to both familiar and unfamiliar communication partners. Levels II–IV describe people who may be slower communicators, to those who have extreme difficulties communicating with either familiar or unfamiliar communication partners. ^3^ The VSS describes the speech production abilities of people with CP. Level I describes people who have typical speech development. Levels II–IV describe people who have imprecise speech, to no understandable speech. * Other cerebral palsy motor type and topography includes more than one predominant motor type and hypotonia. ** Additional neurodevelopmental/psychological disorder includes autism spectrum disorder, depression, anxiety, and post-traumatic stress disorder.

## Data Availability

The data presented in this study are available on request from the corresponding author due to ethical and privacy considerations. The raw qualitative data contain confidential participant information for use in this research study only. Additional consent and ethics approval will need to be obtained prior to the sharing of data.

## Supplementary Materials

The following supporting information can be downloaded at https://www.mdpi.com/article/10.3390/healthcare14020197/s1: Supplementary File S1: Focus group topic guide for MBSR program participants and interview schedule for facilitator. Supplementary File S2: Table S1. Supplemental quotes from focus groups and semi-structured interviews.

## Abbreviations

The following abbreviations are used in this manuscript:

MBSR	Mindfulness-Based Stress Reduction
CP	Cerebral Palsy
