# Supplementary material for: Experiences of a Mindfulness-Based Telehealth Program Modified for Adults with Cerebral Palsy—A Qualitative Study
Source: Healthcare (Basel). 2026 Jan 13;14(2):197. doi: 10.3390/healthcare14020197 (PMC12840942; doi:10.3390/healthcare14020197)
Supplement: Supplementary file 1 [file healthcare-14-00197-s001.zip › healthcare-4060257-supplementary.pdf]

Supplementary File S1

Focus Group Topic Guide for MBSR Program Participants

Overall Experiences of Participant

- What did you like best about the program?
- What did you like least?
- What was the easiest/most challenging part of the program?
- What will you take away from the program?
- Is there anything you would change about the program?

Program Content and Accessibility

- How useful did you find the program content?
- What did you learn from the program?
- Is there anything you would change about the program?
- Were there any parts of the content that made you feel uncomfortable?

- How manageable was it participating in the program?
- Was the program accessible? Was there anything we could do to make it more accessible?

Program Outcomes and Influence of Daily Life

- Do you think having participated in the program will impact on your life moving forward?
- Are there changes you have implemented as a result of the program?
- Do you feel like you have used new skills from this program? Examples?
- Would you recommend this program to others? Why or why not?

Interview Schedule for Facilitator

Overall Experiences of Facilitator

- What did you like best about the program?
- What did you like least?
- What was the easiest/most challenging part of the program?
- Is there anything you would change about the program?
- Would you recommend this program to other facilitators and/or participants? Why or why not?

Perceived Participant Experience

- Were there parts of the program which you felt participants really enjoyed or engaged well with?
- Were there parts of the program which you felt made participants feel uncomfortable/didn't seem to like or engaged well with?
- Did you have any thoughts about what helped participants to benefit most from the program?
- Were there any barriers/challenges which prevented people from getting the most out of the program?

Program Outcomes and Influence of Daily Life

- During the program, did participants mention any changes in their life which may have been related to having participated in the mindfulness program?
- Do you think the program will have an impact on the participants' lives moving forward?

Supplementary File S2

Table S1. Supplemental quotes from focus groups and semi-structured interviews

Themes	Quotes from focus groups and semi-structured interviews	Participant ID
**Theme 1: Learning and creating my mindfulness toolbox**		
Experience of program and feedback on content	It was a good program.. I really enjoyed it.. I think the content was delivered in a way that was really accessible and easy to understand.	P9

Themes	Quotes from focus groups and semi-structured interviews	Participant ID
	I like what we're learning about	P8 (AAC User)
	I just think it's [the MBSR program] something that I've just benefited from so much in a relatively short amount of time....and I found it's made a massive difference…. I'm a little bit disappointed [about the course coming to an end] because I did, I did enjoy it.	P10
	I enjoy mindful breathing in daily life.	P3 (AAC User)
	Well, it's [the MBSR program] taught me a lot of valuable techniques.	P1
Individual preferences	There was some things that suited me, and some things that didn't suit me…. I found that the content really covered a lot, and a lot of bases for all the different people…..the quick ones were pretty good for me, but like, just like, attention span wise.	P7
	Mindfulness is very individual journey and...I guess some things work better for some people than others.	P9
	I think what helped me the most, was the informal mindfulness activities.	P2
	I preferred, sort of, the formal practice of just trying to be within myself.	P4
	I enjoy that in the audio files, the silence in mindful activities. They didn't talk too much.	P3 (AAC User)
	Some people really liked the minding your pain activity, because that was really helpful with pain…..whereas other people that had pain found that there were other activities that worked better for them.	F1
	I definitely did enjoy the formal practices more than the informal.	P5
Challenges	I didn't like the long, like pauses in the meditation.... and I was in a peaceful state like, I just....it just ruined it for me kind of thing.	P6
	I also ha(d) a tendency to forget what we've talked about.	P4
	I thought some of the information was a little bit repetitive.	P9
	Finding ways to fit, like the home practice into my schedule, and like remembering to do it was probably very difficult for me.	P7

Themes	Quotes from focus groups and semi-structured interviews	Participant ID
**Theme 2: Applying mindfulness in daily life**		
Impact on psychological wellbeing	The whole program helped me manage my anxiety better.	P2
	And Participant 3 just said that he "finds that he is calm" (researcher conducting the focus group reading text from chat, in response to how the course has impacted daily life outside of the program).	P3 (AAC User)
	It's [the MBSR program] definitely helped me with, I think, reducing my anxiety a little bit too.	P4
	One of the best things about this program has been that it's just made me more aware of myself and my own mindfulness, just throughout my everyday life in everyday situations... I just know that it's there….So this course has helped me to recognise not only when I have anxiety, but actually stop and think about, "OK, where is this actually coming from?" Like, "what am I anxious about?" And it's allowed me to then, like, acknowledge that feeling and, and really get down to like the root cause of what I'm feeling.	P5
	I just think it's [the MBSR program] made a major impact in like, knowing myself a bit better, and like kind of, yeah, noting those changes in my body that indicate that I'm stressed, or something like that, that I, I never would have picked up on before.	P7
Applications of mindfulness	[I use mindfulness] When I'm driving in car.	P8 (AAC User)
	If you are anxious, or panicky or umm, in an uncomfortable situation and being able to umm, identify those things…. and then have practices to be able to help…things like waiting in a, like, a doctor's waiting room.	P9
	[One participant shared in a group session that] "when I, when I'm sick in bed, I usually get really down, and, but this time I didn't because I was, you know, played with some mindfulness strategies".	F1
Timing of mindfulness practice	I find it easier to concentrate on mindfulness if I'm, if I'm, like, not in the middle of my anxiety, but, but, before I get to the point of an anxiety attack.	P2

Themes	Quotes from focus groups and semi-structured interviews	Participant ID
	There were actually times that I was feeling overwhelmed, and like probably leading into bit of like a panic attack, or something, that would happen later in the day. But I was able to catch it earlier and like, reduce the stimuli.	P7
	I felt I did them [the mindfulness exercises] best when I was in those moments of anxiety....I myself, found it easier when I was in those moments of anxiety. I tried in those times when I wasn't, and it wasn't too bad, it just didn't feel as good a feeling, to me.	P4
	It [the MBSR program] was good because it helped me prevent anxiety, but, um. I found that when I did get anxiety, that would actually trigger me to do the mindful practices.	P5
Other perceived benefits	Before this course, I really struggled to go outside. ...But after this course, because of COVID, but after this course, I'm gonna get involved in, more stuff with my family and my friends. So that's actually really good for me.	P6
	I've had like a lot of stressful situations over the past nine weeks and I think I've handled them a lot better than I previously would have before doing mindfulness.	P7
	I can sleep better, not dream during day.	P8 (AAC user)
**Theme 3: Online together with expert facilitation**		
Accessibility	It [*the online format*] was good for me because I wouldn't have been able to get somewhere. every week.	P9
	Yeah, I thought that was [*the online group]* good. I certainly think it was better than trying to do it in person. Uh, yeah, probably the ease of the access… as opposed to having to get somewhere.	P10
Group learning	A really beautiful active member of the group, and was, they were able to share really lovely support to other people in the group, and also receive some really nice support.	F1
	It was good because we could kind of relate to each other and it kind of like validated some of the experiences….I think it would be really cool if, umm, like the people in the group had like an opportunity to know each other if they wanted	P7

Themes	Quotes from focus groups and semi-structured interviews	Participant ID
	to outside the group…. It's really hard to find people with similar conditions to you and that could be, like, similar life experiences.	
	I like different people's views.	P8 (AAC user)
	I think getting other people's insights and responses to each of the exercises was kind of …..beneficial because someone might say something in a way which would add to the exercises that you hadn't noticed before, or it might spark some kind of insight or some point of conversation. So, I thought that was really nice in our connection.	P9
Expert facilitation	The facilitator that we have was really friendly and inclusive and willing to sort of learn and, you know, be open to ideas.	P9
	The course facilitator was a really good facilitator, in a sense of like, you can see that she's a very calm person and that she actually practices mindfulness herself.....And I think that made it really effective…..like because of my CP, like sometimes if there's too much information being thrown at me, I can forget, or I can.. But The course facilitator's really good at, like, just slowly taking us through things, rather than throwing everything at us.	P5
	Having someone with the expertise to be able to help you, when you are getting stuck, or have particular questions, or are struggling with something, and someone to be able to offer solutions – I found really beneficial.	P10
Facilitator experiences	I would definitely recommend it to other facilitators because I think it is, it is just, like I said the, um, the vicarious joy, the sympathetic joy, it, it is such a treat to facilitate it [the MBSR program].	F1
	I think you need a knowledge of CP...why? Um, well, a lot of the time the participants talk about CP related, um, challenges, I guess, that they're experiencing, you know, so, um, you know, whether it's pain, or whether it's swallowing.	F1
	The biggest thing [facilitators need] is probably being comfortable with facilitating groups, like, um, because, um, it's, there were elements that were tricky.	F1

Themes	Quotes from focus groups and semi-structured interviews	Participant ID
	The mental health stuff, having an understanding of, um, of how anxiety and depression might impact on people, just so you can actually, I guess watch out for, to make sure everybody's travelling well.	F1
	Um, OK, so, probably due to my ignorance, um, prior to the group starting, I was really concerned how I would juggle the dynamics of the group, particularly with people with AAC.	F1
Program recommendations	I think it would be good to at least have, if not doing the course completely again, but at least have just something as a follow up that we can do.	P5
	Maybe a shorter one? if it's a refresher course?	P7
	It would've been easier if we did have a meet-and-greet call, because that way we could tell everyone and what everyone's agreed to. Like, Because I was really nervous to meet everyone.	P2
	Maybe like if you did like a session before the program started where it's like a meet and greet…for everyone…so then we can actually meet every person, and then we can have those conversations [*about AAC etiquette*], like Participant 4's saying... Because I think yeah, cause I think if we had've met Participant 3 before the program actually started, then he could have told us, "Aw, I use this device, here's how I'd like you guys to communicate with me", that kind of stuff.	P5
	Um, OK, so, probably due to my ignorance, um, prior to the group starting, I was really concerned how I would juggle the dynamics of the group, particularly with people with AAC.	F1
	Like maybe if the sessions were a bit shorter because I think they were an hour and a half, something like that. I think that's quite long for people, particularly at the end of a work day on zoom.	P9
	I think an hour [would be a good session duration].	P2
